# Examining the Exercise Dose–Response Using Cardiac Autonomic Activity in Female University Ice Hockey Players

**DOI:** 10.3390/sports13090330

**Published:** 2025-09-16

**Authors:** Maggie L. Peterson, Patrick E. Monforton, Anthony R. Bain, Kevin J. Milne, Andrew S. Perrotta

**Affiliations:** 1 Department of Kinesiology, Faculty of Human Kinetics, University of Windsor, Windsor, ON N9B 3P4, Canada; peter11y@uwindsor.ca (M.L.P.); monfort1@uwindsor.ca (P.E.M.); anthony.bain@uwindsor.ca (A.R.B.); kjmilne@uwindsor.ca (K.J.M.); 2 Centre for Human Performance and Health, University of Windsor, Windsor, ON N9B 3P4, Canada

**Keywords:** heart rate variability (HRV), training load, sport science, athlete monitoring, female athletes

## Abstract

Female university ice hockey players experience elevated and sustained cardiovascular stress during training and competition. There remains limited research on the “exercise dose–response” in female ice hockey players. The purpose of this study was to examine daily and weekly changes in cardiac autonomic activity across a competitive season, and to examine its association with accumulated exercise stress. Twenty-one female ice hockey players wore chest strap heart rate monitors to quantify exercise heart rate dynamics into a training load (TL) metric and time (min) performing high-intensity activity (HIA) during training and competition. Cardiac autonomic activity was expressed as both resting heart rate (RHR) and the root mean squared of successive R-R intervals (rMSSD) and was recorded immediately upon awakening each morning. The association between HRV and both TL (r = −0.420, *p* = 0.058) and HIA (r = −0.420, *p* = 0.058) was observed. The association between RHR and both TL (r = 0.109, *p* = 0.638) and HIA (r = 0.150, *p* = 0.516) was observed. rMSSD fell below the typical error for ~50% of games. In conclusion, HRV demonstrated greater sensitivity to exercise stress than RHR for quantifying the dose–response to on-ice exercise stress.

## 1. Introduction

Ice hockey is a sport that requires elevated and sustained physiological functioning during training and competition [1]. Particularly, the cardiovascular and musculoskeletal systems are heavily relied upon to support the technical, tactical, and physiological demands of competition [1]. Athletes typically participate in on-ice shifts lasting 30–80 s, with a total on-ice time of 15–25 min [2]. Both the central and peripheral nervous system are essential for integrating the physiological response during this time to ensure adequate cardiovascular and musculoskeletal function [3].

University ice hockey programs typically involve a four-to-six-week preseason period, followed by a 26-game competitive season with games played on consecutive days, often providing only a single rest day during the week [4]. The concomitant demands of sport in addition to academic stressor such as exams, assignments, and group projects, has the potential to lead to accumulated fatigue throughout the season [5]. There is a pertinent need for coaches and practitioners to monitor exercise stress and the response stress to optimize athlete health and performance.

The development of valid and reliable forms of wearable technology has allowed practitioners to effectively and efficiently examine the “exercise dose–response” in team sport during training and competition [6]. Previous literature has focused on quantifying the dose of exercise, using external load, in female ice hockey players through recording work completed using metrics such as total distance covered, skating speeds, accelerations, decelerations and changes in direction [7]. There remains a paucity of validated wearable technology utilizing global positioning systems (GPS) or an inertial measurement unit, to quantify external load in ice hockey players due to the indoor setting of an ice hockey rink and the lack of GPS signal acquisition and sensitivity of the inertial movement sensor for recording skating speed [8]. As such, ice hockey arenas are required to install expensive cameras around the rink to capture time-motion analyses for deriving external loads. Recent inquiry towards examining the internal load in female ice hockey players has used exercise heart rate dynamics to quantify exercise stress into a training impulse (TRIMP). This evidence has revealed higher internal training loads in competition compared to training [4], with training loads typically increasing throughout the season [5]. Although this research has provided practitioners with robust evidence for understanding the “dose” of exercise, there remains limited inquiry towards understanding the athlete’s “response” from experiencing the dose.

Cardiac autonomic activity, as demonstrated through heart rate variability (HRV), has briefly been examined for its utility towards quantifying the physiological response of exercise in female ice hockey players. Merrigan et al. (2024) revealed a significant association between alterations in nocturnal HRV and accumulated exercise stress from training and competition. These results demonstrated higher sympathetic activity during the night when exposed to larger exercise stress during the day, also, greater vagal activity after a reduction in exercise stress was experienced [5]. Although this association is promising for practitioners when examining the “exercise dose–response”, nightly monitoring of cardiac activity, and its evaluation is often impractical and unrealistic for most athletes and practitioners [9]. HRV monitoring has emerged as a valuable tool in team sport for tracking training adaptations and recovery status through cardiac autonomic function [10]. HRV offers a sensitive marker for detecting both positive and maladaptive responses to exercise [11], and has been well implemented in female team sports such as soccer and field hockey [12,13]. However, the association between external training load (i.e., GPS) and HRV in female team sport athletes has shown to be less substantial [14,15]. Brief recoding periods for R-R intervals, such as 60 s immediately upon awakening in the morning, enhances athlete adherence and the ability for practitioners to make meaningful decisions when monitoring HRV [16]. Furthermore, the identification of valid and reliable smartphone HRV applications, that integrate with wearable technology, permit rapid HRV analysis [17], supporting evidence-based decision-making around athlete training and recovery.

The purpose of this study was two-fold, (1) to examine daily and weekly changes in cardiac autonomic activity across a competitive university season, as well as differences in HRV between playing year, and (2) to examine the association between indices of cardiac autonomic activity and accumulated exercise stress through both a heart rate derived training load (TL) and time (min) performing high-intensity activity (HIA). In conducting the study, we hypothesized that cardiac autonomic activity would decrease over the season and that significant differences in HRV between playing years would be evident, and that significant inverse associations would be observed between each index of cardiac autonomic activity and both a HR-derived training load and time performing HIA.

## 2. Materials and Methods

### 2.1. Study Design

**Figure 1:**
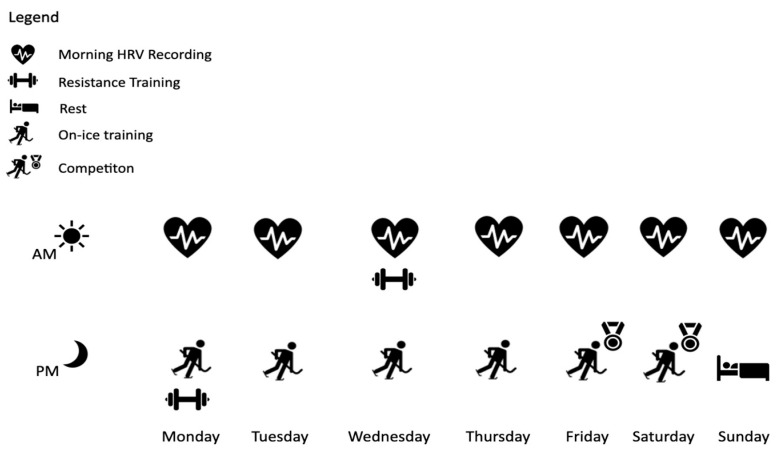
Schematic illustrating the weekly demands of training and competition during the 2023/2024 varsity ice hockey season. The figure also depicts the athlete monitoring schedule, with resting HRV being collected every morning and exercise stress evaluated at all training sessions and competitions.

This was a prospective observational cohort study, examining the “exercise dose–response” using heart rate derived training loads and its interplay with heart rate variability over 145 consecutive days. This study was conducted in Canadian female varsity ice hockey players during the 2023–2024 regular season and playoffs from October 2023 to February 2024. Participants engaged in on-ice team training three to four times per-week from Monday to Thursday and competed in two games every week on Friday and Saturday (Figure 1). One student was responsible for collecting and exporting HRV data, while the second student was responsible for collecting exercise stress data during training and competition. Data sets were exported into a Microsoft Excel^®^ spreadsheet for analysis. Each participant’s morning HRV measurement was recorded in their home upon awakening first thing in the morning. A familiarization phase of seven-days prior to the start of the season, was used to educate participants on how to use equipment and to enhance athlete compliance, a limiting factor when analyzing daily HRV in unsupervised athletes [9]. This study was approved by the Research Ethics Review Board of the University of Windsor and was conducted in accordance with the ethical standard as established in the 1964 Declaration of Helsinki and its later amendment.

### 2.2. Participants

A sample size of ≥17 participants was determined to be acceptable when using a computer software program (G*Power 3.1) [18] that incorporated the conventional α error probability of 0.05 and a power of (1-β error probability) 0.85. Power was determined using previous literature demonstrating the day-to-day variation in rMSSD in female team [19] sport athletes when using a chest strap heart rate monitor and revealed an effect size of 0.61. A total of twenty-one (*n* = 21) female university hockey players with an age of (mean ± SD) 20.4 ± 1.7 years volunteered for this study. Anthropometric measures such as height, body mass, and body fat percentage were assessed using a stadiometer (SECA 213) and the BOD POD (COSMED USA, Concord, CA, USA). Participants had an average height of 166.2 cm (±4.6), body mass 65.3 kg (±7.1), percent body fat of 23.1% (±6.0), fat free mass of 50.0 kg (±3.8) and body mass index of 23.7 kg/m^2^ (±2.5). Only forwards and defence were included in this study. All participants provided written informed consent to participate in all the daily HR monitoring that was conducted at home and during training and competition.

### 2.3. Exercise Stress

Cardiovascular stress was examined during on-ice training and competition using exercise heart rate dynamics and was quantified using Edward’s training load formula [20], a criterion method for establishing an internal training load index [21]. The Edwards training load is a method used to quantify exercise intensity and duration based on personalized heart rate zones. Each participant’s maximum heart rate was determined from the highest value identified during on-ice training and competition during the pre-season period. Edwards method involves recording the time in minutes spent in five predefined heart rate zones, each representing a percentage of each participant’s maximum heart rate (HRmax). These zones range from Zone^#^1 = 50–59%, Zone^#^2 = 60–69%, Zone^#^3 = 70–79%, Zone^#^4 = 80–89% and to Zone^#^5 = 90–100% of HRmax. A training load value is calculated by multiplying the time spent in minutes in each zone by its coefficient (i.e., Zone number) and summing these values. This calculation provides a numerical training load value that is expressed in arbitrary units (AU) [20]. High-intensity activity was defined using time (min) spent in both Zone^#^4 and Zone^#^5. Each participant was allocated a Polar^®^ H-10 chest strap heart rate monitor (Polar, Electro, Oy, Kempele, Finland), equipped to sample at 1000 Hz, and programmed to record at 1-sec intervals. The chest strap was positioned across the sternum over the Xiphoid process, with the electrodes in contact with skin. Depending on each participant’s style of undergarment, the chest strap and heart rate monitor was positioned underneath the elastic band of the sport bra, to maintain proper positioning. Heart rate dynamics were recorded from the beginning of dryland warmup and concluded after the off-ice cool down. Each HR monitor was synched via Bluetooth Smart^®^ technology to a tablet (Apple iPad Pro 4th generation, Apple Inc., Cupertino, CA, USA) that allowed for immediate data collection and storage. Heart rate data collected during training and competition was not used to makeplayers real-time decisions around player playing time, substitutions, or the congruency between desired training loads and actual training loads during training.

### 2.4. Heart Rate Variability & Resting Heart Rate

Cardiac autonomic modulation was examined using HRV. Participants were allocated a chest strap heart rate monitor (Polar H10^®^, Polar, Electro, Oy, Kempele, Finland) equipped to sample at 1000 Hz and validated for recording R-R intervals during rest and exercise for calculating HRV and RHR [22]. Athletes were instructed to leave the equipment bedside to encourage early morning HRV analysis. For standardization, the participants used the same heart rate monitor to capture both training load and HRV. A 60 s ultra-short recording period under spontaneous breathing was used to collect R-R intervals [23] every morning, throughout the study period, immediately upon awakening in the supine position to best represent alterations in cardiac autonomic function as a result of the previous days experienced training load [11]. Each heart rate monitor was synched via Bluetooth^®^ smart technology to the participants individual smartphone for HRV analysis using the Kubios HRV (Version 1.6.6, University of Kuopio, Kuopio, Finland) smartphone application [24]. Participants messaged a screenshot of their morning HRV recording to the graduate student collecting the HRV data. The root mean square of successive differences in R-R intervals (rMSSD) was chosen to assess parasympathetic activity due to its effectiveness in representing resting cardiac vagal modulation [25] and for being robust against respiratory sinus arrhythmia during spontaneous breathing [26]. Additional, HRV time-domain indices such as rMSSD, are not significantly influenced by hormone fluctuations between menstrual cycle phase (*p* = 0.08) [27] or when using oral contraceptives (CV% = 11.1) [28]. An automatic R-R correction algorithm was utilized for correcting ectopic beats or artifacts [29].

### 2.5. Data Analysis

Data analysis was conducted using Microsoft XLStat^®^ statistical software (2020, Addinsoft SARL, Paris, France). Normality of data was examined using a Shapiro–Wilk test. Data sets that were identified to be non-normally distributed were log transformed using the natural log rhythm to help reduce bias caused by any nonuniformity of error.

When examining week-to-week alterations in HRV, a minimum of 3 recordings were required each week for individual data to be included in the mean, which has been shown to be the minimum weekly compliance needed for reasonable assessment of training status in training athletes [30]. Weekly comparison in the mean rMSSD was analyzed using one-way repeated measures ANOVA, with Tukey’s HSD used for post hoc comparison. Additionally, the partial eta squared (η^2^) was calculated to derive the effect size between week-to-week differences in HRV. The smallest worthwhile change in HRV and RHR was determined using the typical error (i.e., the day-to-day change in rMSSD and RHR) and was established using two consecutive complete rest days [19] during the 7-day familiarization period where HRV was assumed to be at its highest during pre-season [31]. The coefficient of variation (CV) for HRV and RHR were calculated (CV = σ/X) to express the typical error, and both were calculated as percentage (CV_HRV_ = 26.2%, CV_RHR_ = 5.3%).

A secondary analysis was performed where participants were categorized into two groups based on their school year; Group#1 (Rookie|Sophomore|Junior) and Group#2 (Seniors). A Mann–Whitney U-test was used to examine differences in rMSSD, RHR, TL, and HIA between groups, while a Rank-Biserial Correlation was calculated to reveal the corresponding effect size. Linear regressions (R^2^) were performed to examine the association between weekly HRV and both training load time spent above anaerobic threshold, as well as weekly RHR and both training load and time spent above anaerobic threshold. A Pearson correlation coefficient (*r*), accompanied by its 95% CI, was used to define the magnitude of the associations. The strength of correlation was categorized as the following: trivial <0.10, small = 0.10–0.29, moderate = 0.30–0.49, large = 0.50–0.69, very large = 0.70–0.89, nearly perfect = 0.9–1.0 [32]. Significance was set to *p* < 0.05.

## 3. Results

Weekly alterations in indexes of exercise stress and cardiac autonomic activity are displayed in Table 1. A one-way ANOVA, with Tukey’s HSD for post hoc comparisons revealed non-significant differences in both weekly rMSSD (df = 20, F = 0.71, *p* = 0.82, η2 = 0.04) and weekly RHR (df = 20, F = 0.78, *p* = 0.74, η2 = 0.04). Within-participant descriptives statistics are provided in Table 2. No significant differences between groups when partitioned by playing year were observed for (mean ± SD); RHR (Group#1 = 62.4 ± 6.3 ‘vs. Group#2 = 61.5 ± 5.6, *p* = 0.82, rB = 0.07), rMSSD (Group#1 = 77.0 ± 27.9 ‘vs. Group#2 = 108.8 ± 49.9, *p* = 0.11, rB = −0.43), TL (Group#1 = 1133.6 ± 281.1 ‘vs.’ Group#2 = 1165.2 ± 293.5, *p* = 0.75, rB = −0.09), and HIA (Group#1 = 96.9 ± 43.2 ‘vs. Group#2 = 116.5 ± 62.7, *p* = 0.51, rB = −0.19).

**Figure 2:**
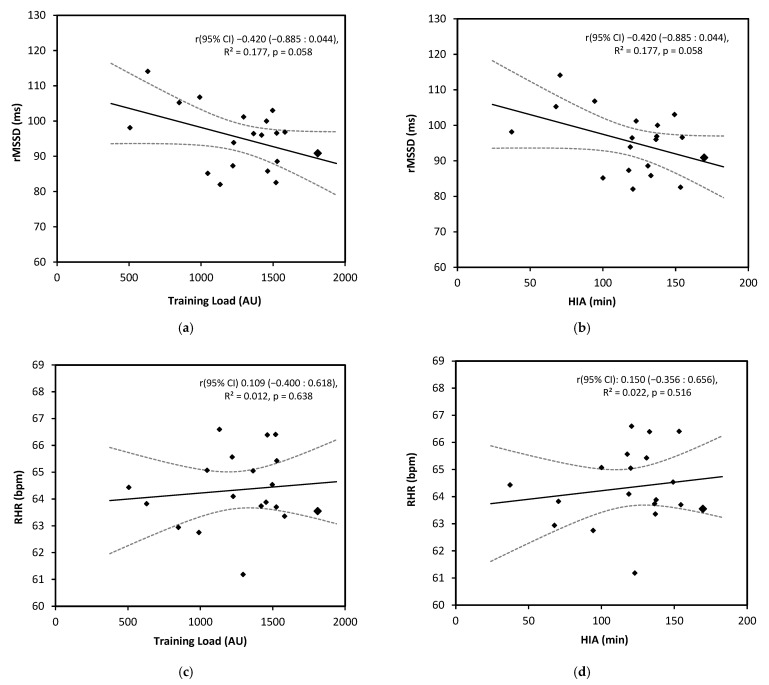
Illustrates the associations between resting cardiac autonomic function and indices of exercise stress. (**a**) Demonstrates the association between weekly mean rMSSD and weekly mean accumulated training load (AU). (**b**) The association between weekly mean rMSSD and weekly mean accumulated time spent performing high-intensity activity (HIA). (**c**) The association between weekly mean RHR and weekly mean accumulated training load (AU). (**d**) The association between weekly mean RHR and weekly mean accumulated time spent performing high-intensity activity (HIA).Each linear regression model provides the trend line as demonstrated using a solid, with the dotted lines representing the corresponding 95% CI.

**Figure 3:**
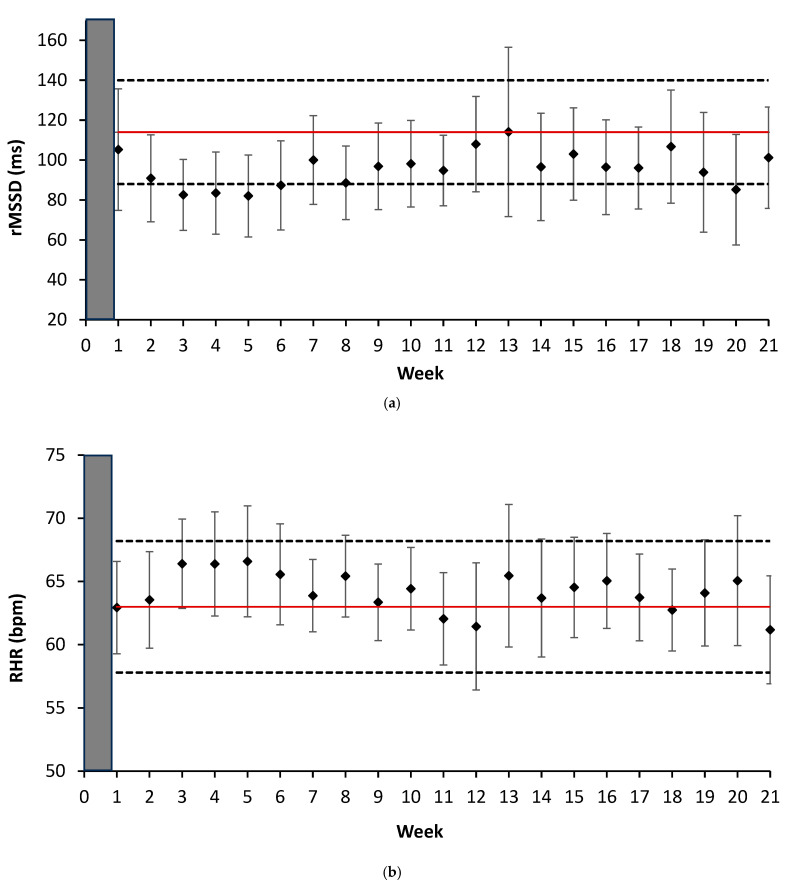
(**a**) Depicts the weekly average (±SD) rMSSD (ms). The red line represents the mean (114 ms) with the dotted lines representing the lower and upper limits of the typical error (CV% = 26.2). (**b**) Displays the weekly average RHR (bpm). The red line represents the mean (63 bpm) with the dotted lines representing the upper and lower limits of the typical error (CV% = 5.3). The grey box represents the familiarization period (i.e., 7-days) prior to week#1 where the typical error for rMSSD and RHR was established. Not all participants were included in the mean values displayed due to a lack of weekly compliance needed (i.e., minimum of 3 recordings).

**Figure 4:**
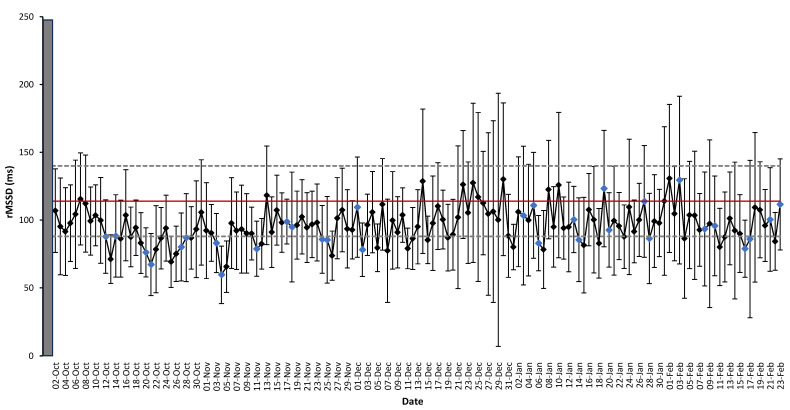
Daily rMSSD is displayed as a mean (±SD) in relation to mean (red line) and the upper and lower bounds (dotted lines) of the typical error (CV% = 26.2). The blue markers indicate competition days throughout the competitive season. The grey box represents the familiarization period (i.e., 7-days) prior to week#1. Not all participants were included in the mean values displayed due to compliance or injury.

Linear regression analyses displaying the association between indices of exercise stress and cardiac autonomic activity are provided in Figure 2. Additional analysis was conducted to examine weekly changes in both rMSSD and RHR across the season in relation to the typical error (Figure 3). Daily alterations in rMSSD were also analyzed in comparison to the typical error. When examining the average rMSSD values on competition days, ~50% of competition days had values below the lower bound of the typical error (Figure 4).

## 4. Discussion

As stated in our hypothesis, inverse associations were observed between cardiac autonomic activity and each index of exercise stress, although these associations were non-significant. Additionally, we failed to observe a linear decrease in cardiac autonomic activity over the complete season; rather, each index revealed an undulation pattern. Furthermore, we observed no significant differences in HRV between playing years.

The current study failed to observe statistically significant differences in weekly HRV or RHR over the course of the season. Given that both cardiac indices are highly individualized and influenced by factors such as fitness level, training intensity, training volume, and age, analyzing differences in the mean may obscure meaningful responses occurring at the individual level, as demonstrated through the corresponding confidences intervals [33]. Although there were no statistical differences in weekly HRV, it is important to consider alterations in physiological function that can be considered meaningful. By examining weekly changes in HRV in comparison to its typical error (HRV_CV_ = 26.2%) we observed reductions in HRV during 6 of the 21 weeks, that resided on or below the coefficient of variation. This data reflects the sensitivity of HRV from accumulated exercise stress and suggests these weeks provided elevated physiological strain or incomplete recovery. When examining the daily HRV response, we observed significant reductions in cardiac vagal modulation during the season as established by the confidence interval. This evidence further supports the utility of monitoring daily HRV in female collegiate ice hockey players [5], and provides practitioners an option that may be more practical for their players, such as ultra-short HRV recordings immediately upon awakening in the morning. Furthermore, morning HRV assessments have demonstrated to be more reflective of previous (i.e., <24 h) exercise stress, as compared to nocturnal analysis, which is highly influenced by the circadian rhythm [9]. In contrast, alterations in weekly RHR remained within its typical error (CV_RHR_ = 5.3%) and displayed trivial and insignificant associations to both TL and HIA, suggesting this index lacks the sensitivity to reflect acute shifts in cardiac autonomic activity in response to exercise stress. As such, the use of RHR alone may prevent practitioners from maladaptive responses in athletes during training, potentially increasing the risk of non-functional overreaching or overtraining if left unaddressed [34]. Therefore, within ice hockey, practitioners are encouraged to incorporate HRV as an athlete monitoring tool to address signs of physiological strain before they lead to accumulated fatigue [34].

Contrary to the findings in this study, recent research conducted in female university ice hockey players demonstrated a continual reduction in HRV over the course of a season. This decrease was attributed to accumulated fatigue from increased training loads. The physiological response to heighted and sustained stress, when examined using HRV, have shown to be compounded across time, whereby cardiac vagal activity can remain suppressed for prolonged periods even after the reduction or removal of stress [11]. The two-week Christmas break in the current study may have provided sufficient time, coupled with a significant reduction in psychophysiological stress, that negated a continued decrease in HRV that was previously observed in the first half of the season. Furthermore, we postulate that the two-week Christmas break mitigated some of the compounding effect, thereby preventing a significant association between HRV and indices of exercise stress to be observed. The findings in the current study further highlight the undulating stress levels and its concomitant effect on HRV in university students over the season [35]. Higher HRV has been associated with improved academic performance in university students [36], further supporting the importance of academic holidays and scheduled rest periods for the wholistic success of students athletes. A secondary analysis examining differences in cardiac autonomic activity between playing years revealed insignificant differences. This approach was taken to account for the effect of age on HRV [37] and is in contrast to previous findings in female collegiate athletes, where rookie volleyball players displayed lower HRV compared to senior players [38]. This difference may be the result of the physiological demands and training style between sports.

Daily analysis of HRV revealed that ∼50% of competition days were accompanied by an rMSSD value below the typical error. Changes in cardiac parasympathetic activity have not been linked to pre-competition anxiety [39]. As such, the large decrements in vagal modulation on competition was likely the result of insufficient recovery from accumulated exercise stress throughout the week. Heightened cardiac vagal modulation has shown to be associated with cardiorespiratory and musculoskeletal performance [40] in addition to improved cognitive decision-making [41]. These attributes are critical for supporting on-ice performance of female ice hockey players during training and competition and reveal the importance for coaches to design periodized weekly training programs to allow for recovery of HRV during competition.

## 5. Limitations

There are some potential limitations that could affect the results of this research. The potential impact of fluctuations in hormones across the menstrual cycle on resting HRV was not examined. However, rMSSD was utilized for its stability between menstrual phases where hormonal alterations do not significantly alter values, [27] and in females taking oral contraceptives [28]. Additionally, participant nutrition and supplement consumption were not tracked throughout the study period, which can have an impact on exercise heart rate dynamics. However, these factors were kept consistent throughout the study. Furthermore, additional stressors outside of exercise and sport, such as academic obligations, travel, and comparison between away and home games may have influenced the results presented. These aspects are encouraged to be utilized in future research examining the “exercise dose–response” using cardiac autonomic activity.

## 6. Conclusions

Weekly HRV, examined using rMSSD, demonstrated greater sensitivity than RHR in detecting alterations in cardiac autonomic function. Although the inverse association between HRV and both TL and time spent performing HIA was insignificant, this relationship should be further explored as extended academic holidays potentially negated the compounded effect to reach statistical significance. Future investigations should emphasize individualized approaches to better capture the “dose–response” to exercise when using HRV and exercise stress in female university ice hockey players.

## Figures and Tables

**Table 1:** Alterations in weekly exercise stress and cardiac autonomic activity.

Week #	rMSSD (ms)	RHR (Bpm)	TL (AU)	HIA (Min)
Week 1	105.3 ± 66.8	62.9 ± 7.8	847.8 ± 228.0	67.75 ± 42.1
Week 2	90.9 ± 47.9	63.5 ± 8.2	1809.1 ± 446.1	169.65 ± 85.1
Week 3	82.6 ± 38.0	66.4 ± 7.3	1519.7 ± 459.2	153.4 ± 73.5
Week 4	85.8 ± 47.1	66.4 ± 9.0	1461.7 ± 503.6	133.1 ± 79.5
Week 5	82.0 ± 45.2	66.6 ± 8.4	1131.3 ± 517.4	120.7 ± 71.7
Week 6	87.3 ± 49.1	65.6 ± 10.1	1219.9 ± 304.4	117.8 ± 56.1
Week 7	100.0 ± 48.9	63.9 ± 5.8	1453.6 ± 501.9	137.6 ± 64.8
Week 8	88.6 ± 40.5	65.4 ± 7.7	1527.4 ± 509.9	130.9 ± 77.1
Week 9	96.9 ± 47.6	63.4 ± 5.8	1582.0 ± 405.7	137.0 ± 68.7
Week 10	98.1 ± 46.3	64.4 ± 6.7	505.5 ± 109.6	37.3 ± 17.1
Week 11	94.8 ± 36.6	62.1 ± 6.4	N/A	N/A
Week 12	108.0 ± 44.8	61.5 ± 6.1	N/A	N/A
Week 13	114.1 ± 70.3	63.8 ± 7.1	628.8 ± 209.9	70.6 ± 50.1
Week 14	96.6 ± 54.1	63.7 ± 5.7	1524.1 ± 640.5	154.6 ± 102.9
Week 15	103.0 ± 50.8	64.5 ± 6.7	1496.2 ± 492.2	149.3 ± 78.7
Week 16	96.4 ± 52.2	65.0 ± 7.2	1364.2 ± 451.3	120.1 ± 71.5
Week 17	96.1 ± 45.1	63.7 ± 6.2	1419.9 ± 348.1	136.6 ± 42.8
Week 18	106.8 ± 62.3	62.8 ± 6.3	989.75 ± 300.0	94.4 ± 41.4
Week 19	93.9 ± 64.0	64.1 ± 7.1	1225.8 ± 522.6	118.9 ± 83.4
Week 20	85.2 ± 53.8	65.1 ± 8.7	1045.875 ± 314.4	100.1 ± 36.9
Week 21	101.2 ± 52.7	61.2 ± 8.6	1294.8 ± 406.8	122.9 ± 48.4

Values are presented as means (±SD). Exercise stress is presented as the accumulated weekly training load (TL) and time (min) spent performing high-intensity activity (HIA). TL is expressed in arbitrary units (AU). N/A = No exercise stress was collected during Christmas break.

**Table 2:** Within-Participant Comparisons in Exercise Stress and Cardiac Autonomic Activity between Playing Year.

School Year	Subject	Playing Position	rMSSD (ms)	RHR (Bpm)	TL (AU)	HIA (Min)
Rookie | Sophomore | Junior	1	F	163.8 ± 22.5	N/A	1688 ± 672.4	287.9 ± 132.6
	2	D	82.1 ± 54.3	67.3 ± 8.7	1084.2 ± 509.9	110.7 ± 45.7
	3	D	60.5 ± 11.9	64.2 ± 4.4	936.4 ± 352.7	102.1 ± 39.0
	4	F	233.0 ± 39.0	47.8 ± 5.1	823.6 ± 381.1	35.9 ± 25.9
	5	F	50.2 ± 15.3	66.1 ± 9.0	1335.8 ± 534.6	113.1 ± 47.1
	6	F	102.8 ± 21.0	58.5 ± 3.8	1251.5 ± 524.0	108.6 ± 51.6
	7	F	80.3 ± 27.7	65.8 ± 8.4	1245.5 ± 495.3	102.7 ± 47.4
	8	F	97.9 ± 37.0	65.0 ± 5.3	1295.7 ± 696.6	126.7 ± 89.8
	9	F	85.5 ± 25.5	63.0 ± 3.1	1098.7 ± 656.1	102.3 ± 62.6
	10	D	89.4 ± 13.7	57.8 ± 4.0	1033.2 ± 629.7	103.6 ± 67.8
	11	F	131.1 ± 17.9	60.5 ± 7.0	1545.8 ± 645.0	156.8 ± 66.1
	12	D	119.9 ± 62.5	59.8 ± 5.6	643.6 ± 319.5	47.4 ± 34.8
Seniors	13	D	88.6 ± 20.2	67.0 ± 6.3	1535.1 ± 634.3	170.7 ± 77.7
	14	F	49.0 ± 16.3	71.9 ± 6.2	716.6 ± 337.1	80.3 ± 35.2
	15	F	108.3 ± 27.0	55.4 ± 7.7	1368.5 ± 427.4	115.0 ± 38.3
	16	D	57.5 ± 11.4	65.9 ± 5.5	10,250.0 ± 545.6	60.9 ± 48.1
	17	F	77.9 ± 21.9	55.7 ± 5.0	784.6 ± 416.7	39.7 ± 21.3
	18	F	68.9 ± 7.2	59.5 ± 6.2	1228.0 ± 501.8	103.2 ± 48.3
	19	D	122.3 ± 20.3	54.6 ± 4.2	1055.2 ± 458.0	100.3 ± 43.3
	20	F	85.1 ± 14.6	63.7 ± 1.7	1059.6 ± 465.4	54.7 ± 29.9
	21	D	34.8 ± 24.9	67.7 ± 12.1	1429.5 ± 545.9	146.5 ± 59.8

Within-participant data (mean ± SD) were calculated using the weekly mean over the 12-week study period. Exercise stress is presented as the accumulated weekly training load (TL) and time (min) spent performing high-intensity activity (HIA). Training load (TL) is expressed in arbitrary units (AU). F = forward, D = defender.

## Abbreviations

The following abbreviations are used in this manuscript:

HRV	HSeart rate variability
RHR	Resting heart rate
rMSSD	The root mean square of successive differences in R–R intervals
HIA	Time spent performing high-intensity activity
TL	Training Loads
AU	Arbitrary Units
